# Epithelioid hemangioma of the penis: case report and review of literature

**DOI:** 10.1186/1752-1947-5-260

**Published:** 2011-06-30

**Authors:** Mohamed Ismail, Stephen Damato, Alex Freeman, Raj Nigam

**Affiliations:** 1 The Royal Surrey County Hospital, Guildford, Surrey, UK; 2 University College London Hospital, London, UK

## Abstract

**Introduction:**

Epithelioid hemangioma is a rare vascular tumor found in the penis. It is essential to avoid misdiagnosis with Peyronie's disease and penile cancer, as management differs significantly.

**Case presentation:**

We present a case of epithelioid hemangioma of the penis in a 50-year-old Caucasian man. We also review the literature to evaluate the incidence of benign vascular anomalies of the penis and their management.

**Conclusions:**

Epithelioid hemangioma of the penis should be considered in the differential diagnosis of patients presenting with painful penile lumps. A thorough histological and immunohistochemical examination is required to make the diagnosis. Optimal management is complete local excision and periodic physical examination for local recurrence.

## Introduction

The differential diagnoses of painful lumps on the penis include Peyronie's disease and penile phlebothrombosis. Epithelioid hemangioma is a rare vascular tumor that is characterized by capillary vessels lined by epithelioid endothelial cells and accompanied by an inflammatory cell infiltrate [1-4]. It should be considered in the differential diagnosis of patients presenting with painful penile lumps. The condition can be easily misdiagnosed as Peyronie's disease or penile cancer. However, the salient feature of penile cancer is painless ulcerative or cauliflower lesions. Making the clinical and radiological distinction is important. We therefore report on this condition and review the literature.

## Case presentation

**Figure 1:**
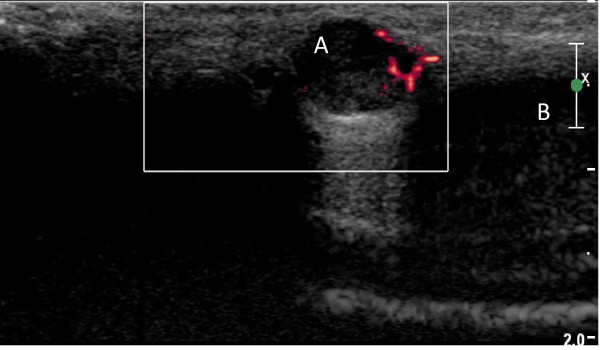
**Ultrasound scan image of the penis**. (A) Ultrasound picture shows the epithelioid hemangioma lesion on the dorsum of the penis. Inherent vascularity is visible within the lesion in red color. (B) Both corpora cavernosa can be seen.

**Figure 2:**
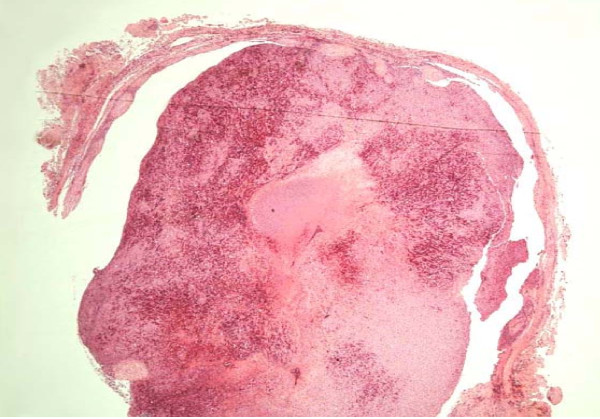
**Low power view of the lesion showing a circumscribed, unencapsulated subcutaneous vascular lesion**.

**Figure 3:**
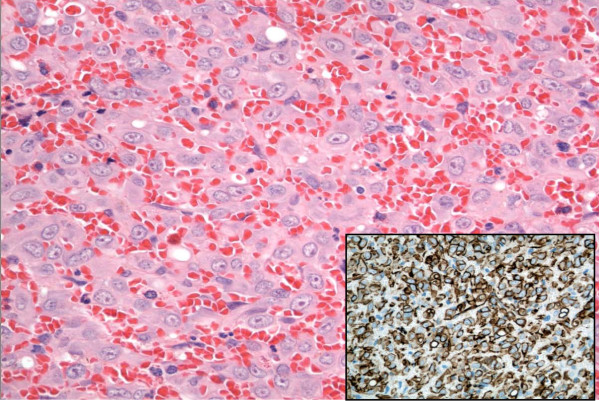
**High power view showing epithelioid cells with abundant eosinophilic cytoplasm**. Immunohistochemistry showed that the epithelioid cells were positive for CD34.

**Figure 4:**
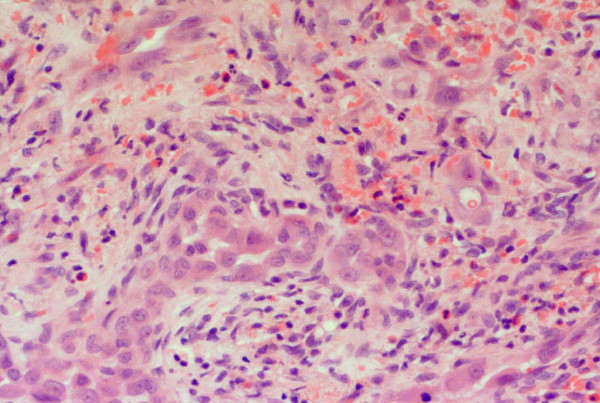
**Periphery of the lesion, containing vessels lined by plump endothelial cells**. Scattered lymphocytes and eosinophils are present.

A 50-year-old Caucasian man presented to our facility with a painful nodule on the dorsum of his penis, which had developed over the last six months. It became more painful over time and interfered with his sexual activity. Our patient reported no erectile dysfunction or penile deviation. His medical history was unremarkable. A physical examination revealed a 5 mm tender, firm nodule at the mid shaft of the penis dorsally. There was no inguinal lymphadenopathy. A penile ultrasound scan demonstrated a well circumscribed lesion (6 × 8 mm) over the dorsal aspect of the penis within the subcutaneous tissue, superficial to the corpus cavernosum (Figure 1). There was inherent blood flow within the lesion. With suspicion of a penile vascular tumor, we performed a local excision of the lesion under general anesthesia. Intra-operatively, the lesion was intimately involved with the neurovascular bundle. Feeding vessels were identified under magnification and were ligated with preservation of the neurovascular bundles. Histopathological examination revealed a well circumscribed, unencapsulated lesion (Figure 2) composed of central sheets of plump epithelioid cells with scattered vessels at the periphery. The epithelioid cells had abundant eosinophilic cytoplasm with large nuclei and distinct central nucleoli (Figure 3). There was no significant cytological atypia and only a few cells in mitosis. There were scattered capillary vessels lined by epithelioid cells at the periphery of the lesion (Figure 4). Immunohistochemical analysis demonstrated strong expression of CD31 and CD34 and no expression of S100 protein or desmin (Figure 3). A pathological diagnosis of epithelioid hemangioma was made. At three months follow-up our patient reported complete resolution of pain and normal erectile function. A physical examination revealed no local recurrence.

## Discussion

Epithelioid hemangioma, first described in 1969, is a rare vascular lesion that typically arises on the head and distal extremities. The pathogenesis is not fully understood and it is unclear whether it represents a benign vascular neoplasm or a reactive process to a preceding trauma [1]. It occurs in mid-adult life and is slow growing, with an average time between appearance and excision of four and a half months [2]. Epithelioid hemangioma does not recur following excision and neither local nor distant metastasis has been reported.

Macroscopically, the epithelioid hemangioma forms an inflammatory red to brown nodule affecting the cutaneous tissue of the head, neck and digits. Microscopically, endothelial cells are arranged in nests surrounded by immature vessels and eosinophil cell infiltrate. The characteristic feature of epithelioid hemangioma is the epithelioid appearance of the endothelial cells, with large nuclei, prominent nucleoli and abundant eosinophilic cytoplasm. The lesion is also characterized by low mitotic index and absent nuclear atypia [5]. Two varieties of epithelioid hemangioma have been identified: the typical and the exuberant (atypical) form. The typical form is surrounded by mature, fully canalized capillary vessels with a defined smooth muscle coat. The exuberant (atypical) form contains aggregates of epithelioid endothelial cells with an indeterminate growth pattern and immature vessels [2]. On immunohistochemical staining, epithelioid hemangiomas are positive for CD31, CD34 and factor VIII-related antigen and negative for keratin and epithelial membrane antigens [6].

Differential diagnoses of epithelioid hemangioma include epithelioid hemangioendothelioma, epithelioid hemangiosarcoma, Kimura's disease and bacillary angiomatosis. Epithelioid hemangioendothelioma is a borderline malignant tumor that has the tendency to recur and metastasize. It is characterized by epithelioid endothelial cells embedded in a hyaline connective tissue matrix with nuclear atypia, and absence of eosinophil infiltrates [7]. Epithelioid hemangiosarcoma is a vascular malignant tumor with aggressive histological and clinical features. Prominent nuclear atypia, high mitotic index and necrosis are usually seen [8]. Kimura's disease is characterized by multiple subcutaneous nodules and is associated with lymphadenopathy and blood eosinophilia. The distinctive feature is the presence of lymphoid follicles with germinal centers [9]. Bacillary angiomatosis is a vascular lesion caused by *Bartonella henselae *bacteria, and affects immune compromised patients. It is characterized by proliferative blood vessels in a myxoid or hyaline matrix and heavy infiltration of neutrophils and bacilli [10].

Management of epithelioid hemangioma of the penis includes taking a detailed history of trauma during sexual intercourse and erectile function. Clinical examination usually reveals a solitary, painful, well circumscribed nodule on the dorsum of the penile shaft with no lymphadenopathy or penile deformity. Clinical investigations include full blood count to exclude eosinophilia, ultrasound scan, color Doppler and MRI scans of the penis and the pelvis if indicated [6]. Treatment is surgical and comprises complete local excision of the tumor with a rim of normal tissue. The tumor is usually associated with the neurovascular bundle on the dorsal aspect of the corpus cavernosum, and care should be taken during dissection to preserve potency. Careful periodic follow-up with physical examination to check for local recurrence is essential.

## Conclusions

This case study demonstrates the importance of making the correct diagnosis of painful penile lumps. Awareness of epithelioid hemangioma is important for urologists and should be included in the differential diagnosis of painful penile lumps. A thorough clinical and radiological evaluation is essential to make the diagnosis. Optimal management appears to be local excision and periodic physical examination for local recurrence.

## Consent

Written informed consent was obtained from the patient for publication of this case report and any accompanying images. A copy of the written consent is available for review by the Editor-in-Chief of this journal.

## Competing interests

The authors declare that they have no competing interests.

## Authors' contributions

MI analyzed and interpreted and wrote the original draft. SD and AF performed the histological examination and were major contributors in drafting the manuscript. RN made a major contribution to writing the manuscript and editing the final version of the paper and figures and was the consultant urologist responsible for the case. All authors read and approved the final manuscript.
